# GAT inhibition preserves cerebral blood flow and reduces oxidant damage to mitochondria in rodents exposed to extreme hyperbaric oxygen

**DOI:** 10.3389/fnmol.2022.1062410

**Published:** 2023-01-10

**Authors:** Ivan T. Demchenko, Hagir B. Suliman, Sergey Y. Zhilyaey, Olga S. Alekseeva, Tatyana F. Platonova, Matthew S. Makowski, Claude A. Piantadosi, Heath G. Gasier

**Affiliations:** ^1^The Duke Center for Hyperbaric Medicine and Environmental Physiology, Duke University Medical Center, Durham, NC, United States; ^2^Sechenov Institute of Evolutionary Physiology and Biochemistry, Russian Academy of Sciences, St. Petersburg, Russia

**Keywords:** anti-inflammation, cerebellum, GABA, hippocampus, mitophagy, mitochondrial biogenesis

## Abstract

Oxygen breathing at elevated partial pressures (PO_2_’s) at or more than 3 atmospheres absolute (ATA) causes a reduction in brain γ-aminobutyric acid (GABA) levels that impacts the development of central nervous system oxygen toxicity (CNS-OT). Drugs that increase brain GABA content delay the onset of CNS-OT, but it is unknown if oxidant damage is lessened because brain tissue PO_2_ remains elevated during hyperbaric oxygen (HBO_2_) exposures. Experiments were performed in rats and mice to measure brain GABA levels with or without GABA transporter inhibitors (GATs) and its influence on cerebral blood flow, oxidant damage, and aspects of mitochondrial quality control signaling (mitophagy and biogenesis). In rats pretreated with tiagabine (GAT1 inhibitor), the tachycardia, secondary rise in mean arterial blood pressure, and cerebral hyperemia were prevented during HBO_2_ at 5 and 6 ATA. Tiagabine and the nonselective GAT inhibitor nipecotic acid similarly extended HBO_2_ seizure latencies. In mice pretreated with tiagabine and exposed to HBO_2_ at 5 ATA, nuclear and mitochondrial DNA oxidation and astrocytosis was attenuated in the cerebellum and hippocampus. Less oxidant injury in these regions was accompanied by reduced conjugated microtubule-associated protein 1A/1B-light chain 3 (LC3-II), an index of mitophagy, and phosphorylated cAMP response element binding protein (pCREB), an initiator of mitochondrial biogenesis. We conclude that GABA prevents cerebral hyperemia and delays neuroexcitation under extreme HBO_2_, limiting oxidant damage in the cerebellum and hippocampus, and likely lowering mitophagy flux and initiation of pCREB-initiated mitochondrial biogenesis.

## Introduction

Under normal conditions the partial pressure of oxygen (PO_2_) in the brain is ~30–40 mmHg in rats and humans but breathing hyperbaric oxygen (HBO_2_) increases it in proportion to the inspired PO_2_ (Demchenko et al., 2005; Ponce et al., 2012). As consequence, oxidants accumulate and disrupt neurotransmission leading to convulsions resembling epilepsy, neuronal trauma and necrosis, and death, i.e., central nervous system oxygen toxicity (CNS-OT; Piantadosi and Tatro, 1990; Oury et al., 1992; Demchenko and Piantadosi, 2006; D'Agostino et al., 2007). For HBO_2_ therapy where the inspired PO_2_ is between 2.0–2.9 atmospheres absolute (ATA) with air intervals, the incidence of seizures is 0.02–0.6% (Smerz, 2004; Costa et al., 2019). In divers who use closed-circuit oxygen rebreathers, the incidence of CNS-OT (signs/symptoms and seizures) is 2.5–7% (Butler and Thalmann, 1986; Arieli et al., 2006). Limited knowledge of how brain cells respond to increased PO_2_ has prevented expansion of HBO_2_ indications and oxygen exposure profiles, warranting further study.

Following the discovery of γ-aminobutyric acid (GABA) and its inhibitory function on neuroexcitation in the mammalian CNS (Roberts and Frankel, 1950; Basemore et al., 1957), Wood and Watson (1962) postulated that GABA was involved in oxygen seizures. The hypothesis was supported by data showing that the concentration of brain GABA was reduced in pharmacologically induced convulsions due in part to glutamic acid decarboxylase (GAD) inhibition, and GABA administration offered some level of protection against seizures in animals and humans (Killam and Bain, 1957; Roberts, 1959; Baxter and Roberts, 1960; Gulati and Stanton, 1960). Wood et al. (Wood and Watson, 1963, 1964; Wood et al., 1967, 1969) determined the following: whole brain GABA levels decrease as a function of inspired PO_2_ above 3 ATA and is due to GAD inhibition, the rate of decline in brain GABA content is related to oxygen seizure latencies, and pretreatment with GABA decreases oxygen seizure incidence, severity and mortality. In the United States, GABA is not prescribed for epilepsy, leading us to test the efficacy of FDA approved antiepileptic drugs in mice exposed to HBO_2_ at 5 ATA (Demchenko et al., 2019). Of these drugs, GABA enhancers demonstrated the best efficacy in delaying HBO_2_ seizures. Because seizures cause DNA damage (Cantafora et al., 2014), astrocyte reactivity (Chipres-Tinajero et al., 2021), and increased permeability to the blood–brain barrier (Bargerstock et al., 2014), maintaining brain GABA levels may lessen oxidant damage in brain cells. If, however, cerebral blood flow (CBF) responses to HBO_2_ are unabated, PO_2_ will increase and promote oxidant brain injury. This has not been studied in HBO_2_.

Our objective was to measure GABA’s role in cerebrovascular control and oxidant brain injury. Moreover, since HBO_2_ damages mitochondria and activates mitochondrial biogenesis (Balentine, 1974; Gutsaeva et al., 2006), we measured activation signaling pathways of mitophagy and mitochondrial biogenesis that are linked to oxidants and inflammation. Focus was placed on the cerebellum and hippocampus due to their susceptibility to oxygen-induced neuronal injury (Balentine, 1982; Gutsaeva et al., 2006). Our approach was to reduce GABA reuptake from the synaptic cleft by inhibiting GABA transporters (GATs) with nipecotic acid (NPA) or tiagabine (TGB). Nipecotic acid is a nonselective inhibitor of brain GATs (1–3), whereas TGB selectively inhibits GAT1 (Nielsen et al., 1991; Kragler et al., 2005). Our hypothesis was that in extreme HBO_2_, GAT inhibition lessens oxidant injury by delaying neuroexcitation independently of changes in CBF. Given less injury, stimulation of mitochondrial turnover signaling would be reduced.

## Materials and methods

All procedures were approved by the Duke University Institutional Animal Care and Use Committee (IACUC) and the Ethical Review Board of the Sechenov Institute of Evolutionary Physiology and Biochemistry Russian Academy of Sciences. Rats were used for cerebrovascular control experiments because physiological stress responses are similar to humans (Goutianos et al., 2015). Mice were used to study oxidant brain injury because their sensitivity to oxygen resembles humans (Marks, 1944).

### Experimental protocol in anesthetized rats

Experiments were performed at the Duke Center for Hyperbaric Medicine and Environmental Physiology. Male Sprague Dawley rats weighing 317–367 g (Charles River Laboratories) were anesthetized with IP urethane (750 mg/kg) and α-chloralose (250 mg/kg), placed on a heating pad with rectal thermometer, and ventilated mechanically with 30% oxygen using a small animal respirator (Edco Scientific Inc.). Catheters were inserted into the femoral artery and vein for blood pressure monitoring and drug delivery, respectively. Heads were positioned in a stereotaxic frame (David Kopf Instruments), and two stainless steel screws were placed into the left and right parietal cortexes for electroencephalogram (EEG) recording. CMA 11 microdialysis probes (0.24 mm, CMA Microdialysis AB) and platinum needle electrodes (100 μm) were inserted into the caudate-putamen (striatum) using a micromanipulator and stereotaxic coordinates (Paxinos and Watson, 2007). Microdialysis probes were continuously perfused with artificial cerebral spinal fluid (aCSF) at a rate of 1 μl/min using a CMA microinjection pump (Carnegie Medicine). The platinum electrodes were used for measuring CBF by the hydrogen clearance method (Demchenko et al., 1998). Electrocardiogram (ECG) electrodes were placed bilaterally under the chest skin for measuring heart rate (RR interval). Anesthesia was maintained throughout the experiments by administering one-fourth the initial doses, and pancuronium bromide (500 μg/kg) was provided to inhibit involuntary respiratory movements. The breathing gas was changed to 100% oxygen after baseline measurements, and rats remained at 1 ATA or were compressed to 3, 5, or 6 ATA at a rate of 0.6 ATA/min. Temperature, relative humidity and CO_2_ were maintained at 23–25°C, 60 and < 0.05%, respectively.

For measurement of interstitial amino acids, rats were exposed to 1 (*n* = 8), 3 (*n* = 7), 5 (*n* = 8), and 6 ATA (*n* = 20) oxygen for 75 min. After a 60 min stabilization period with aCSF infusion, baseline dialysate samples were collected in vials containing 1% perchloric acid before and every 15 min during exposures using a CMA 142 Microfraction Collector (CMA Microdialysis AB). In another group of rats exposed to 6 ATA oxygen (*n* = 8), aCSF was changed to a mixture of aCSF + 70 μM NPA (MilliporeSigma, 656356) after baseline sampling. Amino acids (aspartate, GABA, glutamate, glutamine, glycine, and serine) in the dialysate were measured by o-phthalaldehyde derivatization and HPLC with electrochemical detection (Donzanti and Yamamoto, 1988).

For measurement of cardio-and cerebrovascular responses in HBO_2_, rats (*n* = 16) were exposed to 6 ATA oxygen for 75 min. After a 60 min stabilization period and baseline recording of heart rate, arterial blood pressure, EEG and striatal CBF, rats were injected with 7 μl of aCSF (*n* = 8) or aCSF + TGB (MilliporeSigma, 1667280) in 5% DMSO (0.34 μmol, *n* = 8) into the lateral ventricle 30 min before HBO_2_. Measurements were performed every 15 min in HBO_2_.

### Experimental protocol in conscious rats

Experiments were performed at the Sechenov Institute of Evolutionary Physiology and Biochemistry. Male Sprague Dawley rats weighing 301–349 g were procured from Pushkino Animal-Breeding Facility. One week prior to HBO_2_, rats were anesthetized with IP pentobarbital (50 mg/kg). A cannula was inserted into the lateral ventricle for drug delivery and secured with acrylic dental cement and two stainless steel anchor screws (EEG in a subset of animals). In 14 rats, PE-50 tubing containing 0.9% NaCl + 2.5% glucose + 300 IU/ml heparin was inserted into the right carotid artery toward the aorta, secured, and tunneled subcutaneously to the back of the neck. Rats were provided penicillin (30,000 IU/kg/day), and the catheter was flushed daily with saline. On experimental days, 7 μl of aCSF (*n* = 14), TGB (0.34 μmol, *n* = 11), or NPA (1.1 μmol, *n* = 14) was administered over 2 min with a Hamilton micro syringe 30 min before exposure to HBO_2_ at 5 ATA. Rats were placed in a pressure chamber (100 L) and the oxygen pressure was increased to 5 ATA at a rate of 1 ATA/min. Temperature, relative humidity, and CO_2_ levels were maintained similarly as above. The catheterized rats (aCSF, *n* = 7 and TGB, *n* = 7) were lightly restrained in a hammock for arterial blood pressure and heart rate (calculated from arterial blood pressure pulse) monitoring. Animals were monitored with a camera and exposures were terminated upon the appearance of seizures, EEG spikes in the lightly restrained rats, or up to 90 min.

### Experimental protocol in conscious mice

Experiments were performed at the Duke Center for Hyperbaric Medicine and Environmental Physiology. Male C57BL/6 J mice (*n* = 57) aged 8–10 weeks (~25–30 g) were procured from The Jackson Laboratory. Mice were assigned to air vehicle (*n* = 11), air TGB (*n* = 11), HBO_2_ vehicle (*n* = 17), and HBO_2_ TGB (*n* = 18). Mice were administered vehicle (0.9% sodium chloride) or TGB (4.8 mg/kg) IP in a volume of 5 μl per g body weight 30 min before air or HBO_2_ exposures. This TGB dose was selected based on our previous work showing extended seizure latencies by a factor of ~ 3 over controls (Demchenko et al., 2019). Up to five mice at a time were placed individually in plastic cylinders (22 cm in length and 11.5 cm in diameter). The cylinders were flooded with 100% oxygen for 5 min before compression to 5 ATA oxygen at 0.75 ATA per min. The chamber temperature was maintained between 23 and 25°C. After 30 min at 5 ATA, mice were decompressed to sea level at 0.75 ATA per min.

Because brain GABA levels peak ~40 min after IP injections (Fink-Jensen et al., 1992), air and HBO_2_ mice were staggered by 30 min to ensure euthanasia (isoflurane) and brain harvest was completed quickly. Brains were flash frozen in liquid N_2_ or sectioned (sagittal), placed in tissue embedding cassettes and 10% formalin for 24 h before transferring to 70% ETOH and refrigerating at 4°C. Samples were sent to the Duke Substrate Services Core & Research Support for paraffin embedding and slide preparation. In some mice after brain harvest, blood was collected from the abdominal aorta using a 1 ml syringe with a 23-gauge needle and placed in 0.6 ml serum separator tubes (BD Vacutainer®). After 30 min at room temperature, samples were centrifuged at 6,000 *g* for 90 s. Serum was transferred to Eppendorf vials and frozen at −80°C. Serum S100 calcium-binding protein B (S100B) was quantified using an ELISA assay (LSBio, LS-F5980).

### Immunofluorescence

Slides from mice that matched group mean seizure latencies in HBO_2_, and random air mice were incubated with primary antibodies diluted in phosphate-buffered saline (PBS) overnight, washed × 2 in PBS for 10 min, incubated in secondary antibodies diluted in PBS for 1 h, and washed × 2 in PBS for 5 min. Primary antibodies included ATP5A (ATP synthase F1 subunit α; Abcam, Ab14748), citrate synthase (GeneTex, GTX110624), focal adhesion kinase family interacting protein of 200 kD (FIP200, Invitrogen™, PA528563), cAMP response element-binding protein (CREB, Santa Cruz, sc-186), phosphorylated (p)-CREB at Ser-133 (Santa Cruz, sc-7978), glial fibrillary acidic protein (GFAP, Booster Immunoleader, MA1045), heme oxygenase 1 (HO-1, Enzo, ADI-SPA-896F), LC3A/B (LC3-II; Cell Signaling, 4108), PTEN-induced kinase 1 (PINK1, Abcam, ab23707), and 8-hydroxy-2’deoxyguanosine (8-OHdG, Santa Cruz, Sc66036). Antibodies were diluted 1:400 except LC3A/B (1:100). Goat anti-rabbit IgG (Alex Fluor™ 488) and goat anti-mouse IgG (Alex Fluor™ 568) secondary antibodies were purchased from ThermoFisher Scientific and diluted 1:400. All incubations were performed at room temperature. Coverslips were mounted using ProLong™ Gold Antifade Mountant with DAPI (Invitrogen™, P36935) and stored at 4°C until imaging. From three animals/group, six regions were imaged at 60 −100× magnification. Multichannel images were captured from each section using a Nikon Eclipse 50i microscope with a DS-Ri2 color CMOS camera and Nikon Plan Fluor objectives. The signal intensity in collected images were compared to the signal of negative controls and used to determine exposure times and prevent false positives. Nikon NIS-Elements AR software v5.30 was used for quantification.

### RT-qPCR

Total RNA was isolated from brain tissue using RNAqueous-4 PCR kits (ThermoFisher Scientific). Following DNase treatment (4 units for 1 h at 37°C) and inactivation, cDNA was prepared with a high-capacity cDNA archive kit (Applied Biosystems). The following TaqMan® primers were purchased from ThermoFisher Scientific: autophagy-related protein 9A (*Atg9a*, Mm01264420_m1), *Fip200* (Mm00456545_m1), HO-1 (*Hmox1*, Mm00516006_m1), nuclear respiratory factor 1 (*Nrf1*, Mm00447996_m1), peroxisome proliferator-activated receptor γ coactivator 1-α (*Ppargc1a,* Mm01208836_g1), superoxide dismutase 2 (*Sod2*, Mm00449726_m1), mitochondrial transcription factor A (*Tfam*, Mm00447485_m1), and 18S ribosomal RNA (RN18S, Mm03928990_g1). All reactions were completed on a StepOnePlus Real-Time PCR System (Applied Biosystems) for 40 cycles. Data were analyzed using the Fold change assay (DataAssist, v3.01, Applied Biosystems) after normalizing to 18S in each sample and control (air vehicle).

### Data monitoring and statistical analysis

Heart rate, arterial blood pressure, striatal CBF, and EEG were recorded and analyzed using WinDaq software and DI-200 data acquisition hardware (DATAQ Instruments) or with LabScribe 2 software on iWorx IX-228/S hardware (iWorx Systems). Interstitial amino acid levels measured at 1, 3, 5, and 6 ATA oxygen were analyzed using a one-way repeated measures ANOVA. Linear regression was used to determine the relationship between GABA and inspired PO_2_. A two-way repeated measures ANOVA was used to determine the effects of exposure (air and HBO_2_), treatment (aCSF and NPA or TGB), and interaction (exposure × treatment) on interstitial GABA levels, heart rate, mean arterial blood pressure, and striatal CBF in anesthetized and conscious rats exposed to 6 and 5 ATA oxygen, respectively. Seizure latencies in rats and mice were compared using a *t*-test and one-factor ANOVA. For all measures in conscious mice, a two-factor ANOVA was used to determine the effects of exposure (air and HBO_2_), treatment (vehicle and TGB) and interaction (exposure × treatment). A Bonferroni *t*-test was used in *post hoc* analysis. For immunofluorescence data, variances were unequal (Brown-Forsythe) for all measurements except for GFAP in the hippocampus and HO-1 in the cerebellum. In these instances, analysis was performed on transformed data (log or square root). Data were analyzed with SigmaPlot 14.0 (Systat Software Inc.). Values are means ± SD. A *p* < 0.05 was considered statistically significant.

## Results

### Inspired PO_2_ modulates brain interstitial GABA levels

**Figure 1:**
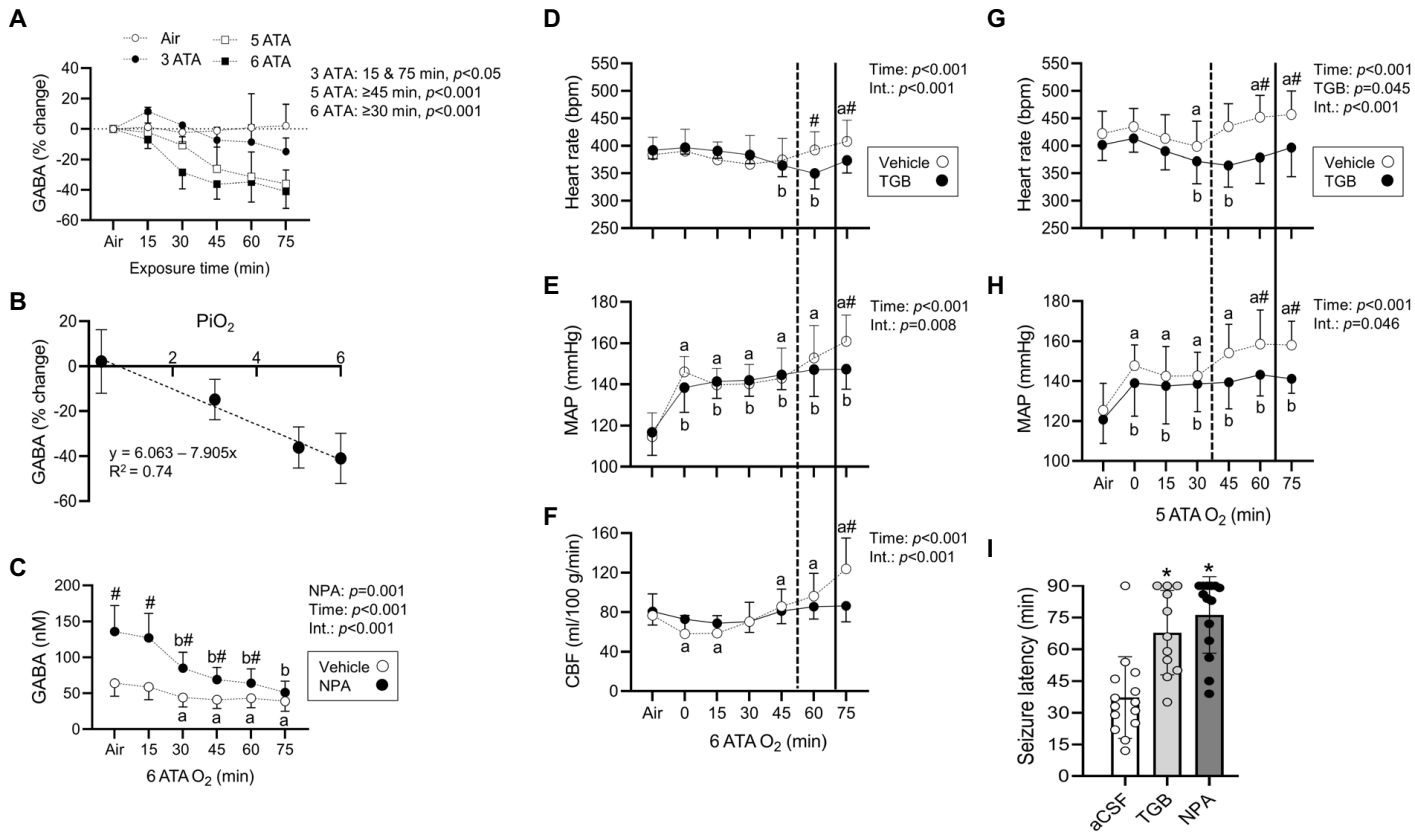
Striatal interstitial γ-aminobutyric acid (GABA) levels, cardiovascular and cerebral blood flow (CBF) responses in rats exposed to extreme HBO_2_. **(A)** Changes in the concentration of GABA measured before and every 15 min in air, 3, 5, and 6 atmospheres absolute (ATA) oxygen (*n* = 7–8/group). **(B)** The relationship between changes in GABA levels (75 min to Air) and the inspired PO_2_ (PiO_2_). **(C)** Concentration of GABA measured before and every 15 min in HBO_2_ at 6 ATA in rats perfused with aCSF (CON) or aCSF + NPA (70 μM) into the caudate putamen (*n* = 8/group). **(D–F)** Heart rate, mean arterial blood pressure (MAP), and CBF were measured in anesthetized rats before and during exposure to 6 ATA oxygen (*n* = 8–10/group). **(G,H)** Heart rate and MAP were measured in conscious rats before and during exposure to 5 ATA oxygen (*n* = 7–8/group). In both experiments, rats were injected with aCSF (CON) or aCSF + TGB in 5% DMSO (0.34 μmol/7 μl) into the lateral ventricle 30 min before HBO_2_. **(D–H)** Vertical dotted (aCSF) and bold (TGB) lines represent mean seizure latencies determined from EEG recordings. **(I)** Mean seizure latency in conscious rats injected with aCSF, aCSF + TGB in 5% DMSO (0.34 μmol/7 μl), or aCSF + NPA in 5% DMSO (1.1 μmol/7 μl) into the lateral ventricle 30 min before HBO_2_ at 5 ATA (*n* = 11–14/group). Values are means ± SD. Time dependent changes in ^a^aCSF and ^b^NPA or TGB groups, *p* < 0.05. ^#^Between group differences, *p* < 0.05. ^*^Significantly different from aCSF rats, *p* < 0.001.

Changes in striatal interstitial GABA and other amino acids (aspartate, glutamate, glutamine, glycine, and serine) were measured in anesthetized rats exposed to HBO_2_ at 6 ATA for 75 min (Table 1). The striatum is the largest structure in the basal ganglia and central for coordinating behavior and motor function, and contains both GABA_A_ and GABA_B_ receptors (Lacey et al., 2005; Mathew et al., 2010; Girault, 2012). The concentration of GABA progressively declined in HBO_2_ to 41% of pre-exposure values. After 75 min, serine and glutamine levels decreased by 26 and 17%, respectively. Aspartate, glycine and glutamate content remained stable in HBO_2_. We also measured the effect of inspired PO_2_ from 0.3 to 6 ATA on extracellular GABA levels (Figure 1A). At 3 ATA, GABA content initially increased and fell thereafter, reaching significance at 75 min. Increasing the inspired PO_2_ to 5 and 6 ATA led to faster and greater declines in GABA levels, mirroring the responses in brain PO_2_ (Demchenko et al., 2005).

**Table 1:** Interstitial amino acids measured in the striatum of rats exposed to HBO_2_ at 6 ATA.

	Air	15 min	30 min	45 min	60 min	75 min	Time, *p* value
Aspartate, μM	0.14 ± 0.10	0.16 ± 0.12	0.17 ± 0.13	0.17 ± 0.12	0.17 ± 0.11	0.16 ± 0.11	0.317
GABA, nM	64 ± 27	61 ± 27	46 ± 20^**^	42 ± 19^**^	41 ± 17^**^	38 ± 16^**^	<0.001
Glutamate, μM	0.99 ± 0.38	0.97 ± 0.12	0.91 ± 0.32	0.90 ± 0.39	1.01 ± 0.30	0.87 ± 0.36	0.595
Glutamine, μM	25 ± 8	26 ± 11	24 ± 10	24 ± 9	23 ± 9	21 ± 9^**^	<0.001
Glycine, μM	0.86 ± 0.23	0.87 ± 0.35	0.92 ± 0.44	0.85 ± 0.29	0.77 ± 0.26	0.73 ± 0.30	0.046
Serine, μM	0.62 ± 0.43	0.59 ± 0.48	0.63 ± 0.57	0.54 ± 0.45	0.52 ± 0.43	0.46 ± 0.42^*^	0.012

Values are means ± SD. Data (*n* = 8–12/amino acid and time point) were analyzed with a one-way repeated measures ANOVA and Bonferroni *t*-test. For glycine, *post hoc* testing revealed no significant differences. Significantly different from Air: ^*^p < 0.05, ^**^p < 0.001.

When GABA content (75 min from air) is plotted as a function of inspired PO_2_, a linear decrease is observed (Pearson’s *r* = 0.86, *p* < 0.001; Figure 1B). To understand the effect of inhibiting GABA transport on interstitial GABA levels, NPA or aCSF were continuously delivered to the striatum before and in HBO_2_ at 6 ATA (Figure 1C). NPA led to an initial more than 2-fold increase in interstitial GABA levels over rats infused with aCSF, however, levels declined to control values by the end of exposures. These data support an inhibitory effect of increased PO_2_ on extracellular striatal GABA production (Wood and Watson, 1964; Wood et al., 1967; Gasier et al., 2017).

### TGB prevents cerebral hyperemia in HBO_2_

In HBO_2_ at 5–6 ATA, a seizure is accompanied by a rise in heart rate, a secondary increase in mean arterial blood pressure and increased CBF (Demchenko et al., 2014). To determine if GABA alters these responses, we administered TGB or aCSF to anesthetized and conscious rats exposed to 6 and 5 ATA of oxygen for 75 min, respectively. In anesthetized rats, TGB prevented tachycardia, hypertension and cerebral hyperemia, and delayed the appearance of EEG spikes by 18 ± 13 min compared to controls (*p* = 0.013; Figures 1D–F). In addition, electrical discharges were present in only 25% of rats treated with TGB compared to 80% in controls. In conscious rats, TGB was equally efficacious in preventing tachycardia and hypertension (Figures 1G,H), and in extending seizure onset by 31 ± 19 min compared to controls (*p* < 0.001). To determine if GABA reuptake is primarily through GAT1, we compared seizure latencies in conscious rats administered aCSF, TGB, or NPA and exposed to 5 ATA oxygen for 90 min (Figure 1I). While NPA increased mean seizure latencies by 9 ± 18 min over TGB, mean group differences were not significant. These data indicate that inhibiting GABA reuptake mainly through GAT1 prevents cerebral hyperemia and delays seizures in extreme HBO_2_.

### TGB lessens oxidant injury in the cerebellum and hippocampus

**Figure 2:**
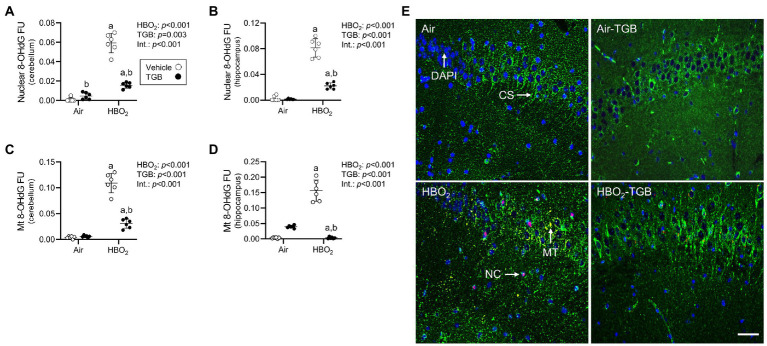
Nuclear and mitochondrial DNA oxidation in mice exposed to extreme HBO_2_. Mice were pretreated with 0.9% NaCl or TGB (4.8 mg/kg body weight) 30 min before exposure to air or 5 ATA oxygen for 30 min. **(A–D)** Quantification of nuclear and mitochondrial 8-OHdG in the cerebellum and hippocampus. **(E)** Representative images from the hippocampus. 8-OHdG (red), citrate synthase (CS, green), and DAPI (blue). 8-OHdG merged with DAPI indicates nuclear oxidation (NC). 8-OHdG merged with citrate synthase indicates mitochondrial oxidation (MT). Scale bar = 100 μm. Main effect of ^a^HBO_2_ and ^b^TGB, *p* < 0.001.

**Figure 3:**
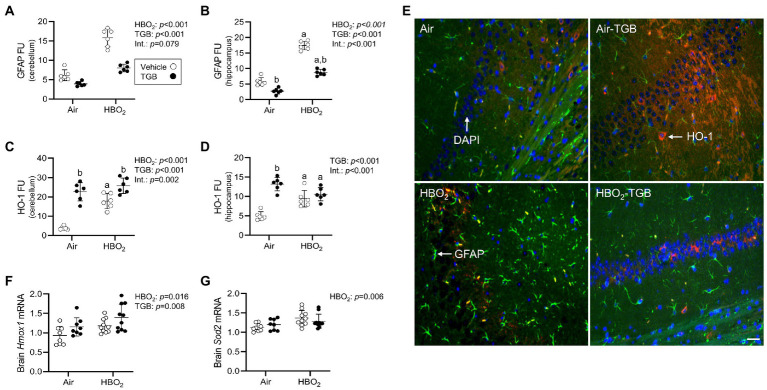
Reactive astrocytes and antioxidant responses in mice exposed to extreme HBO_2_. Mice were pretreated with 0.9% NaCl or TGB (4.8 mg/kg body weight) 30 min before exposure to air or 5 ATA oxygen for 30 min. **(A–D)** Quantification of glial fibrillary acid protein (GFAP) and heme oxygenase-1 (HO-1) in the cerebellum and hippocampus. **(E)** Representative images from the hippocampus. HO-1 (red), GFAP (green), and DAPI (blue). Scale bar = 20 μm. **(F,G)** *Hmox1* and mitochondrial superoxide dismutase (*Sod2*) mRNA expression in brain homogenates (*n* = 8–10/group). Main effect of ^a^HBO_2_ and ^b^TGB, *p* < 0.05.

To explore if GAT1 inhibition protects the brain from oxidant injury, mice were pretreated with 0.9% NaCl or TGB before exposure to 5 ATA oxygen for 30 min. This profile caused 82% of control mice to exhibit motor convulsions at a mean time of 11.6 ± 9.4 min, whereas TGB reduced this to 44% (mean latency 23.0 ± 9.5 min; *p* < 0.001). We assessed oxidant brain injury by measuring nuclear and mitochondrial DNA oxidation and astrocyte reactivity using 8-OHdG and GFAP, respectively (Cantafora et al., 2014; Chipres-Tinajero et al., 2021). HBO_2_ caused significant nuclear and mitochondrial DNA oxidation (Figures 2A–E) and increased astrocyte reactivity (Figures 3A,B,E) in the cerebellum and hippocampus. As an indicator of blood–brain barrier integrity (Kanner et al., 2003), we measured serum S100B and observed a 28% increase in HBO_2_ exposed mice independent of treatment (Air, 48 ± 5 pg./ml vs. HBO_2_, 61 ± 9 pg./ml; *p* < 0.001). TGB pretreatment decreased the oxidant injury in the cerebellum and hippocampus.

A compensatory response to increased oxidant stress is transcriptional activation of antioxidant and anti-inflammatory genes mediated by the nuclear factor erythroid 2-related factor 2 (Nrf2) transcription factor (Zhang and Hannink, 2003; Nguyen et al., 2005; Suliman et al., 2017). After HBO_2,_ HO-1 protein expression in the cerebellum and hippocampus were increased (Figures 3C–E), as were *Hmox1* and *Sod2* mRNA expression in brain homogenates (Figures 3F,G). TGB further increased HO-1 protein expression in the cerebellum. This indicates that GAT1 inhibition reduces oxidant brain damage and activates HO-1, but does not abolish it since DNA oxidation, astrocyte reactivity, and serum S100B remained elevated over air-control mice.

### TGB influences oxidant-stress mediated mitophagy and mitochondrial biogenesis signaling in HBO_2_

**Figure 4:**
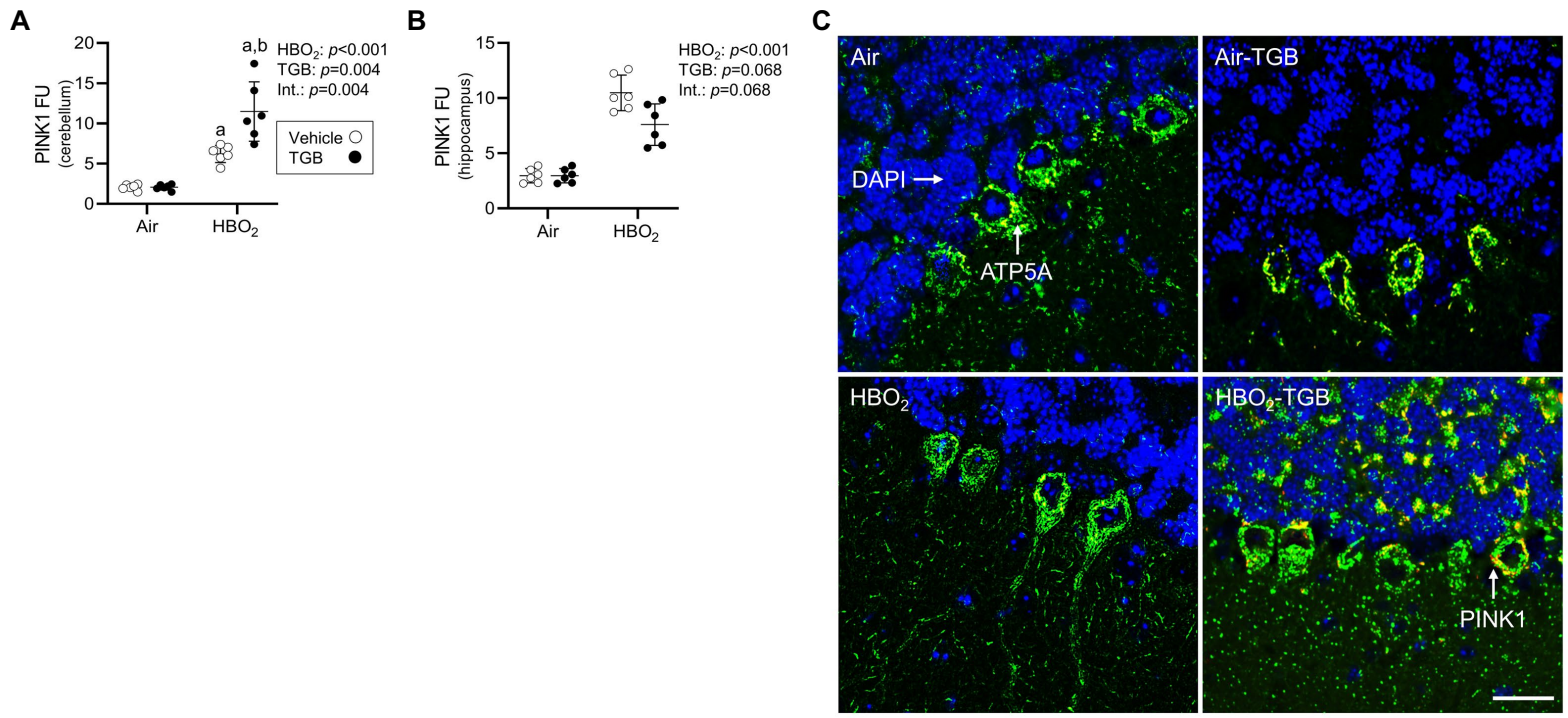
Mitophagy initiation in mice exposed to extreme HBO_2_. Mice were pretreated with 0.9% NaCl or TGB (4.8 mg/kg body weight) 30 min before exposure to air or 5 ATA oxygen for 30 min. **(A,B)** Quantification of PTEN-induced kinase 1 (PINK1) in the cerebellum and hippocampus. **(C)** Representative images from cerebellum. PINK1 (red), ATP synthase F1 subunit α (green) and DAPI (blue). Scale bar = 100 μm. Main effect of ^a^HBO_2_ and ^b^TGB, *p* < 0.05.

**Figure 5:**
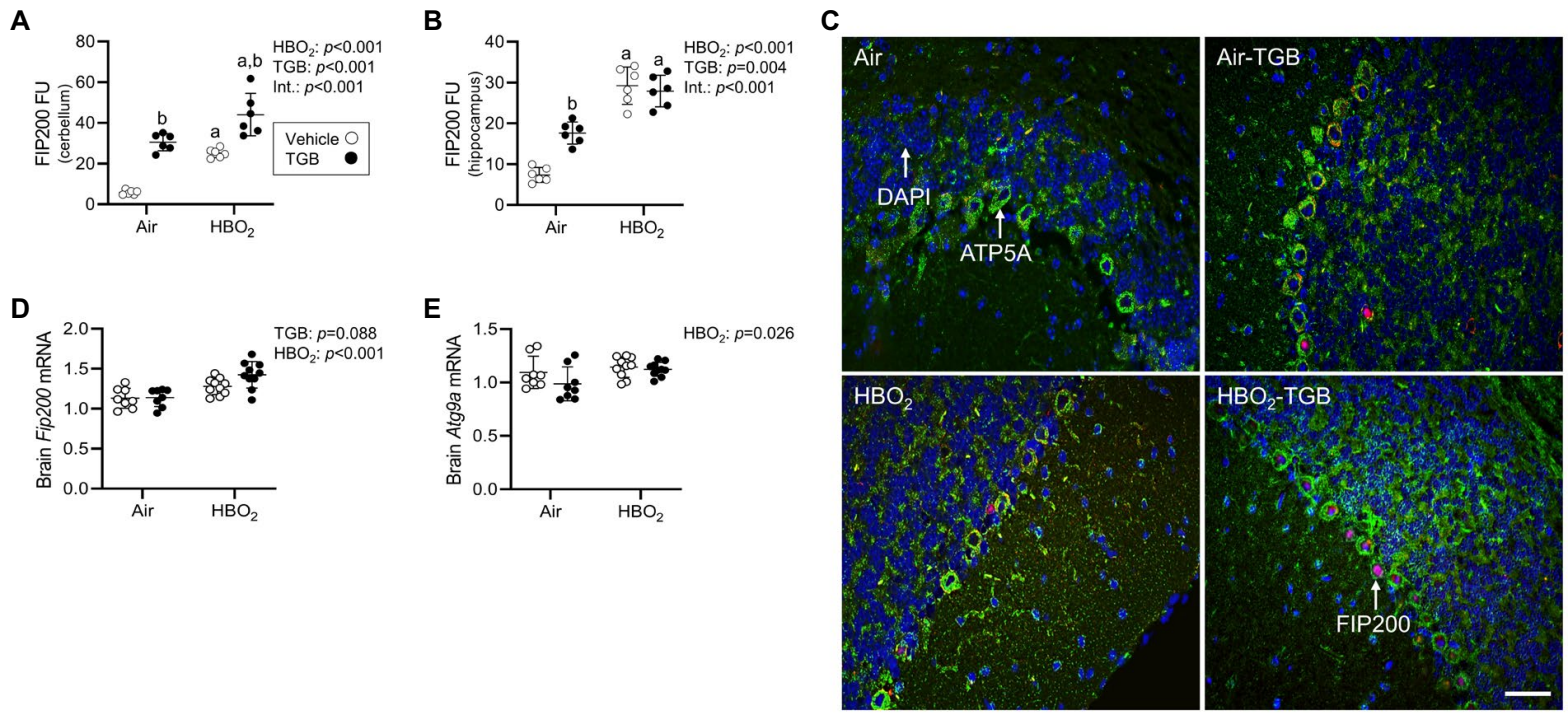
Phagophore formation in mice exposed to extreme HBO_2_. Mice were pretreated with 0.9% NaCl or TGB (4.8 mg/kg body weight) 30 min before exposure to air or 5 ATA oxygen for 30 min. **(A,B)** Quantification of focal adhesion kinase family interacting protein of 200 kD (FIP200) in the cerebellum and hippocampus. **(C)** Representative images from cerebellum. FIP200 (red), ATP synthase F1 subunit α (green) and DAPI (blue). Scale bar = 20 μm. **(D,E)** *Fip200* and autophagy-related protein 9A (*Atg9a*) mRNA expression in brain homogenates (*n* = 8–10/group). Main effect of ^a^HBO_2_ and ^b^TGB, *p* < 0.01.

**Figure 6:**
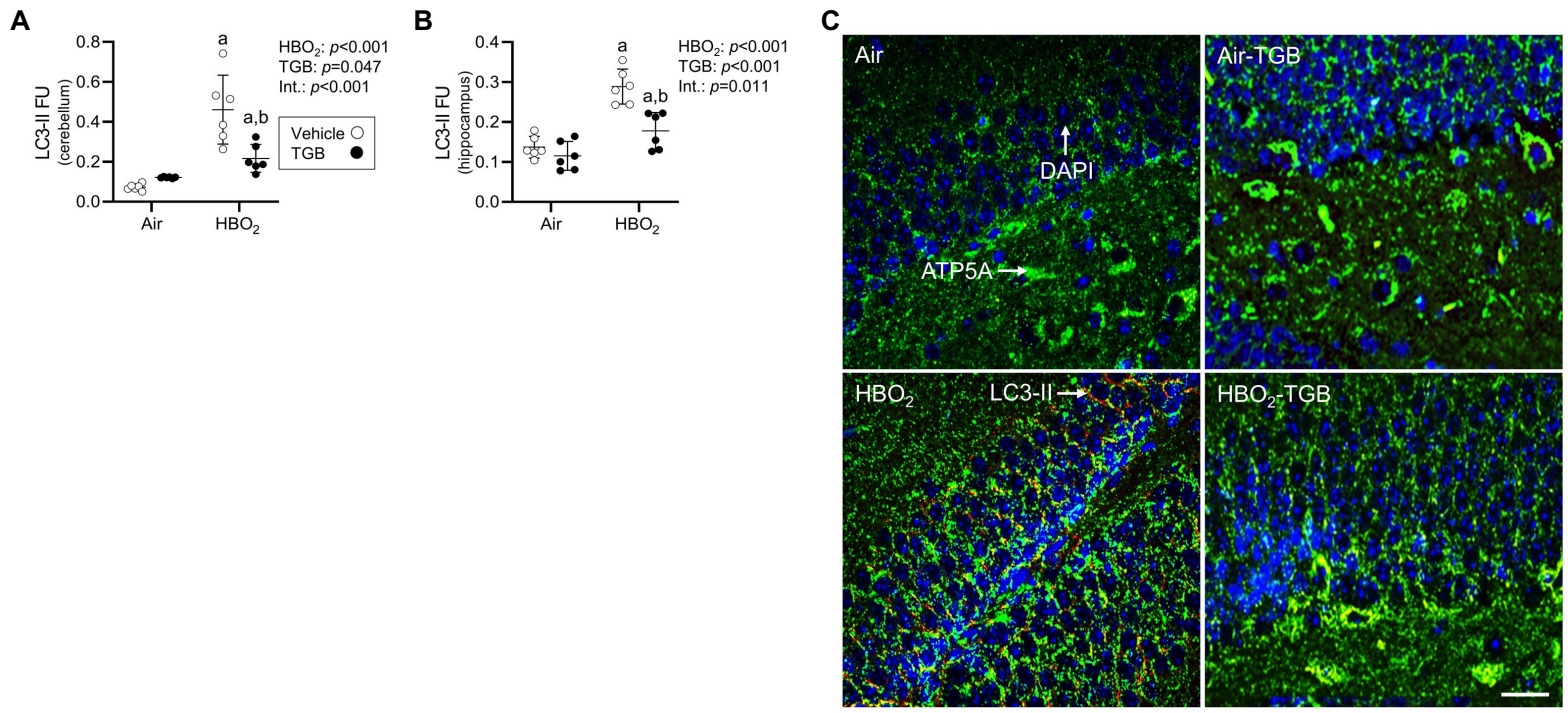
Autophagosome formation in mice exposed to extreme HBO_2_. Mice were pretreated with 0.9% NaCl or TGB (4.8 mg/kg body weight) 30 min before exposure to air or 5 ATA oxygen for 30 min. **(A,B)** Quantification of LC3-II in the cerebellum and hippocampus. **(C)** Representative images from hippocampus. LC3-II (red), ATP synthase F1 subunit α (green) and DAPI (blue). LC-II merged with ATP5A indicates mitophagy. Scale bar = 100 μm. Main effect of ^a^HBO_2_ and ^b^TGB, *p* < 0.05.

In the same mice, we explored activation of mitophagy and mitochondrial biogenesis. During cellular stress and mitochondrial damage, an initial response is for PINK1 to accumulate on the outer mitochondrial membrane (Narendra et al., 2010), resulting in recruitment and phosphorylation of ubiquitin and the E3 ubiquitin ligase Parkin, a critical step in activation of PINK1/Parkin-dependent mitophagy (Matsuda et al., 2010; Kane et al., 2014). HBO_2_ enhanced PINK1 expression in the cerebellum and hippocampus and TGB led to a further increase in the cerebellum (Figures 4A–C). Phagophore formation is required to recognize damaged mitochondria, a FIP200 dependent process that includes configuration of the UNC-51-like kinase (ULK1) initiation complex (Hara et al., 2008). HBO_2_ increased FIP200 in the cerebellum and hippocampus (Figures 5A–C) and mRNA in brain homogenates (Figure 5D). HBO_2_ also increased *Atg9a* mRNA in brain homogenates (Figure 5E), which is required for organized PINK1/Parkin dependent mitophagy and phagophore growth (Lahiri and Klionsky, 2021). TGB increased FIP200 in both regions, and augmented levels above HBO_2_ controls in the cerebellum. The expanded phagophore is coated with LC3 that is converted to LC3-I and conjugated to LC3-II, an autophagosome marker that fuses with lysosomes (Kabeya et al., 2000). HBO_2_ increased LC3-II in mitochondria, and TGB attenuated this response (Figures 6A–C). These data indicate HBO_2_ activates the classical PINK1 dependent mitophagy pathway, and TGB reduces LC3-II accumulation despite an increase in activation signaling primarily in the cerebellum.

**Figure 7:**
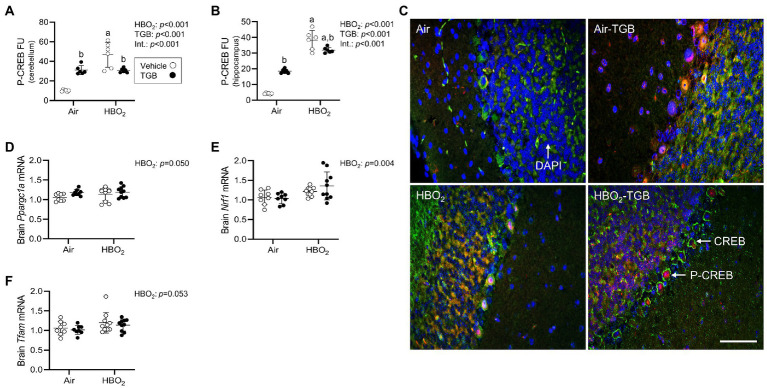
Initiation of mitochondrial biogenesis signaling in mice exposed to extreme HBO_2_. Mice were pretreated with 0.9% NaCl or TGB (4.8 mg/kg body weight) 30 min before exposure to air or 5 ATA oxygen for 30 min. **(A,B)** Quantification of phosphorylated cAMP response element-binding protein at Ser-133 (pCREB) in the cerebellum and hippocampus. **(C)** Representative images from cerebellum. pCREB (red), CREB (green), and DAPI (blue). Scale bar = 20 μm. **(D–F)** Peroxisome proliferator-activated receptor gamma coactivator 1-α (*Ppargc1a*), nuclear respiratory factor 1 (*Nrf1*) and mitochondrial transcription factor A (*Tfam*) mRNA expression in brain homogenates (*n* = 8–10/group). Main effect of ^a^HBO_2_ and ^b^TGB, *p* < 0.05.

In order to maintain a healthy mitochondrial volume density, mitochondrial biogenesis must ensue. To determine if this process is stimulated by our HBO_2_ protocol, we measured levels of p-CREB at Ser-133. Phosphorylated CREB transcriptionally activates peroxisome proliferator-activated receptor gamma coactivator 1-α (PGC-1α; Sheng et al., 2012). PGC-1α and nuclear NRF1 transcriptionally co-activate TFAM, which is required for mitochondrial DNA replication and transcription (Wu et al., 1999). After HBO_2_, pCREB levels increased in the cerebellum and hippocampus (Figures 7A–C), along with *Ppargc1a*, *Nrf1*, and *Tfam* mRNA in brain homogenates (Figures 7D–F). TGB increased pCREB protein expression independent of exposure and reduced the HBO_2_-induced increase in both regions. These data imply HBO_2_ stimulates pCREB mediated biogenesis signaling and TGB attenuates the response.

## Discussion

**Figure 8:**
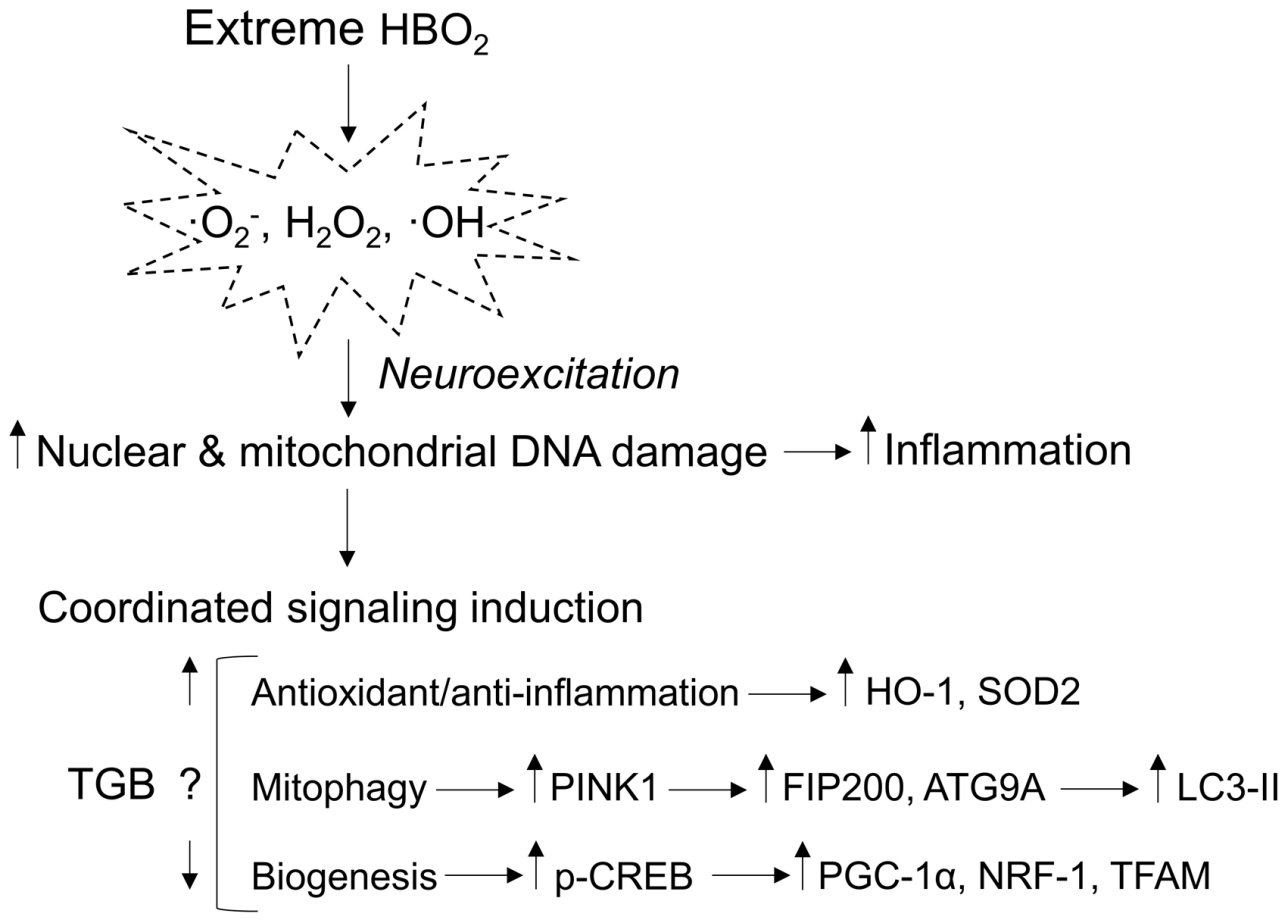
Mitochondria-related signaling responses to extreme HBO_2_. HBO_2_ increases brain tissue PO_2_ leading to amplified production of oxidants, i.e., superoxide (·O_2_^−^), hydrogen peroxide (H_2_O_2_), and hydroxyl (·OH) that target nuclear and mitochondrial DNA resulting in inflammation. The brain immediately activates antioxidant and anti-inflammation defenses that are linked to mitophagy and mitochondrial biogenesis programs. TGB prevents cerebral hyperemia and a further increase in brain tissue PO_2_ that delays neuroexcitation. The result is less oxidant damage and inflammation that may reduce mitophagy flux and mitochondrial biogenesis signaling. HO-1, heme oxygenase 1 (antioxidant/anti-inflammation); SOD2, manganese-dependent superoxide dismutase (mitochondrial antioxidant); PINK1, PTEN-induced kinase 1 (mitophagy initiation); FIP200, focal adhesion kinase family interacting protein of 200 kD (phagophore formation); ATG9A, autophagy-related protein 9A (phagophore growth); LC3-II, conjugated microtubule-associated protein 1A/1B-light chain 3 (autophagosome-lysosome fusion); p-CREB, phosphorylated cyclic-AMP responsive element binding protein (mitochondrial biogenesis); PGC-1α, peroxisome proliferator-activated receptor γ coactivator 1-α (mitochondrial biogenesis); NRF-1, nuclear respiratory 1 (transcriptional activation of TFAM); and TFAM, mitochondrial transcription factor A (mitochondrial DNA transcription, replication, and packaging).

γ-aminobutyric acid’s main CNS function is to bind post-synaptic GABA receptors that facilitate ion flux and hyperpolarization, depressing excitatory postsynaptic potentials. In HBO_2_ at or above 3 ATA, GABA production is reduced by oxidant production and modification of GAD, leading to seizures (Wood and Watson, 1963; Wood et al., 1969; Gasier et al., 2017). GABAergic drugs delay seizure onset in HBO_2_, but its effect on oxidant brain injury is unknown since brain tissue PO_2_ may not be affected by GABA reuptake. To address this, we exposed anesthetized and conscious rats and mice to 5 and 6 ATA oxygen, which causes rapid development of CNS-OT and oxidant brain injury (Balentine, 1982; Demchenko et al., 2014). Our experiments show two novel findings: First, GAT inhibition increases extracellular GABA content, and prevents cerebral hyperemia. Second, a single exposure to HBO_2_ that causes oxidant brain injury activates antioxidant and anti-inflammation, mitophagy and mitochondrial biogenesis signaling (Figure 8). TGB attenuates oxidant damage and modifies these responses. Either directly or indirectly, these findings show that GABA’s function extends beyond inhibitory neurotransmission in extreme HBO_2_.

Our microdialysis data indicate that interstitial GABA levels in the striatum fall in relation to inspired PO_2_. This is consistent with Wood et al. (1969) who measured GABA in whole brain homogenates from rats exposed to HBO_2_ from 4 to 7.5 ATA for 20 min. The mechanism for reduced GABA levels is inhibition of GAD activity caused by a reduction in presynaptic neuronal release and negative feedback inhibition and/or S-nitrosylation of GAD65 (Wood et al., 1967; Green et al., 1987; Gasier et al., 2017). The decrease in extracellular glutamine 75 min into HBO_2_ is not easily explained because of the complexity in the glutamine-glutamate/GABA cycle. In the brain, glutamine is synthesized only in astrocytes from glutamate and ammonia by glutamine synthetase, an endergonic reaction (Walls et al., 2015). Glutamine is shuttled to glutamatergic and GABAergic neurons where it is reconverted to glutamate/GABA. In times of increased NAD^+^-to-NADH, glutamate can undergo oxidative deamination, yielding α-ketogluatarate and ammonia (McKenna and Ferreira, 2016). The reaction is catalyzed by glutamate dehydrogenase in mitochondrion. Ammonia levels are reported to increase in the striatum of rodents after HBO_2_ seizures, and NADH oxidation in the rat brain precedes EEG spikes in extreme HBO_2_ (Mayevsky et al., 1974; Mialon et al., 1992, 1995). While not statistically significant, extracellular glutamate content decreased by 140 nM from 60 to 75 min in HBO_2_. Because striatal glucose consumption increases during EEG spikes in rats exposed to HBO_2_ (Torbati and Lambertsen, 1983), our data may imply oxidative deamination increased to support energy demand and led to decreased glutamine levels. Alternatively, the reduction in extracellular glutamine may be due to glutamine synthetase inhibition or increased neuronal uptake to replenish glutamate (Kanamori and Ross, 2011). The reduction in extracellular serine can be explained by conversion to D-serine or glycine, both of which serve as co-agonists with glutamate to activate neuroexcitatory N-methyl-D-aspartate (NMDA)-type glutamate receptors (Traynelis et al., 2010). Glycine is also an inhibitory neurotransmitter and decreased over time in HBO_2_ as a result of decreased substrate (serine), hydroxymethyltransferase activity, or postsynaptic receptor binding (Murtas et al., 2020).

Inhibition of GABA reuptake by neurons and astrocytes with NPA resulted in a large increase in extracellular GABA content. Even with continuous infusion, however, the magnitude of decline was greater with NPA (−166%) than controls (−64%). GABA levels were similar after 75 min, at about seizure onset. The dose of NPA used here, 70 μM, is above the IC_50_ for inhibiting GABA reuptake in cortical neurons (12 μM), astrocytes (16 μM), and striatal synaptosomes (3.6 μM; Mantz et al., 1994; Falch et al., 1999). Plausibly, the rapid decline in extracellular GABA may reflect maximal GABA receptor binding vs. glial reuptake because NPA was infused throughout HBO_2_ exposures.

In HBO_2_, CBF is regulated by three principal factors: CO_2_, nitric oxide (NO), and neurovascular coupling. Here, injecting an IC_50_ dose of TGB (47 nM) into the lateral ventricle of rats before HBO_2_ maintained cerebral vascular resistance and prevented cerebral hyperemia. We can only exclude alveolar CO_2_ as a factor since the rats’ ventilation is maintained during the experiments (Demchenko et al., 1998). Changes in neurovascular coupling, however, may explain why cerebral hyperemia was prevented with TGB. In rat and human studies, CBF and cerebral glucose utilization are reduced when treated with the GABAergic agonist’s muscimol and vigabatrin, respectively (Kelly and McCulloch, 1983; Spanaki et al., 1999). Active electrical discharges were not present in 75% of the rats treated with TGB, supporting intact neurovascular coupling. However, EEG spikes were present in other rats that exhibited different CBF patterns, i.e., one increased by 19% (seizure latency 57 min) and the other decreased by 9% (seizure latency 49 min). Clearly, further study will be needed in this area.

In conscious rats, a single 30 min exposure to 5 ATA oxygen immediately increases neuritic degeneration and mitophagy in the spinal cord (Balentine, 1982). In the brain, this level of PO_2_ only leads to mild neuronal degeneration within the hippocampus and cerebellum that is not detectable until days after HBO_2_ (Gutsaeva et al., 2006). The mouse is more susceptible to CNS oxygen toxicity than rats (Wood et al., 1967), as evidenced by a mean seizure latency of ~ 37 min in rats compared with ~ 12 min in mice in this study. Seizure latency correlates with a rise in oxidant production that continues to increase with exposure time (Atochin et al., 2003). The results reported here include nuclear and mitochondrial DNA oxidation and astrocytosis in the cerebellum and hippocampus, and modest opening of the blood brain barrier. The oxidant production triggered activation of antioxidant and anti-inflammatory enzyme systems that are integrated with mitophagy and mitochondrial biogenesis (Piantadosi et al., 2011; Suliman et al., 2017). The signal is hydrogen peroxide (H_2_O_2_) generated spontaneously or by the dismutation of superoxide (·Q_2_^−^) *via* SODs and is increased in extreme HBO_2_ (Piantadosi and Tatro, 1990). The extent and time course of mitochondrial turnover and its influence on cell repair remain unknown.

Maintaining vascular resistance and extending seizure latencies should reduce oxidant production, but not eliminate it because brain tissue PO_2_ is increased 15-fold in HBO_2_ at 5 ATA and rises to over 1,000 mmHg during the appearance of EEG spikes (Demchenko et al., 2005). Indeed, TGB reduced nuclear and mitochondrial DNA oxidation and astrocytosis in the cerebellum and hippocampus. Greater HO-1 protein expression may explain the reduction in astrocyte activation found during HBO_2_. In support of this, Godai and Moriyama (2022) reported pregabalin or gabapentin increased HO-1 and decreased GFAP mRNA expression in the spinal dorsal horn of mice with nerve injury. However, the effects were abolished when the HO-1 inhibitor tin protoporphyrin IX was used. While these drugs do not bind GABA receptors, they do effect GABAergic transmission. In the hippocampus, TGB prevented mitochondrial DNA oxidation and normalized HO-1 protein expression in HBO_2_, suggesting that mitochondrial damage was less than in the cerebellum, thus reducing initiation of mitophagy. The pattern of PINK1, FIP200 and LC3-II in the hippocampus supports this notion. However, TGB also reduced the expression of LC3-II in the cerebellum despite increased PINK1 and FIP200 protein expression. Reduced LC3-II may also indicate protein degradation within lysosomes. Thus, TGB may reduce oxidant damage and mitochondrial turnover requirements. Reduction of excess pCREB protein expression in HBO_2_ mice treated with TGB strengthens this assertion. The increase in pCREB in TGB treated control mice may be due increased GABA, which is reported to increase pCREB levels (Auger et al., 2001). Also, pCREB has other functions that include anti-inflammation and neurogenesis (Jagasia et al., 2009; Li et al., 2018). The increased HO-1 and reduced GFAP protein levels in TGB treated mice along with the limited duration of our experiments favors an anti-inflammatory mechanism.

In summary, we show for the first time that GAT inhibition modifies the cerebrovascular responses to extreme HBO_2_ that serve to protect against oxidant brain injury. HBO_2_ initiates mitophagy and pCREB mediated mitochondrial biogenesis signaling. The antiepileptic drug TGB maintains CBF and delays neuroexcitation, which perhaps lowers the requirements for mitochondrial turnover. The time course for brain injury repair, and whether TGB shortens recovery time remain unknown. This study expands our knowledge of the therapeutic function of GABAergic signaling that could be extended to other conditions such as epilepsy, stroke, and brain trauma.

## Data availability statement

The original contributions presented in the study are included in the article/supplementary material; further inquiries can be directed to the corresponding author.

## Ethics statement

The animal study was reviewed and approved by The Duke University Institutional Animal Care and Use Committee (IACUC) and the Ethical Review Board of the Sechenov Institute of Evolutionary Physiology and Biochemistry Russian Academy of Sciences.

## Author contributions

ID and CP developed the experimental design. ID, CP, and HG directed the overall research. ID, HS, SZ, OA, TP, MM, and HG performed the experiments. ID, HS, MM, and HG analyzed the data and prepared the figures. All authors contributed to the article and approved the submitted version.

## Funding

The work was supported by the Office of Naval Research, Award #: N00014-18-1-2702 (CP and HG), the Russian Science Foundation, Award #: 22-25-00539 (ID), and the National Institutes of Health, Award #: T32 HL160494-01 (MM).

## Conflict of interest

The authors declare that the research was conducted in the absence of any commercial or financial relationships that could be construed as a potential conflict of interest.

## Publisher’s note

All claims expressed in this article are solely those of the authors and do not necessarily represent those of their affiliated organizations, or those of the publisher, the editors and the reviewers. Any product that may be evaluated in this article, or claim that may be made by its manufacturer, is not guaranteed or endorsed by the publisher.
